# Associations Between Sentinel Lymph Node Biopsy and Complications for Patients with Ductal Carcinoma In Situ

**DOI:** 10.1245/s10434-018-6410-0

**Published:** 2018-03-07

**Authors:** Brigid K. Killelea, Jessica B. Long, Weixiong Dang, Sarah S. Mougalian, Suzanne B. Evans, Cary P. Gross, Shi-Yi Wang

**Affiliations:** 1 Cancer Outcomes, Public Policy, and Effectiveness Research (COPPER) Center, Yale Cancer Center and Yale University School of Medicine, New Haven, CT USA; 2 Department of Surgery, Yale University School of Medicine, New Haven, CT USA; 3 Department of Chronic Disease Epidemiology, Yale University School of Public Health, 60 College Street, New Haven, CT 06520 USA; 4 Section of Medical Oncology, Department of Internal Medicine, Yale University School of Medicine, New Haven, CT USA; 5 Department of Therapeutic Radiology, Yale University School of Medicine, New Haven, CT USA; 6 Section of General Internal Medicine, Department of Internal Medicine, Yale University School of Medicine, New Haven, CT USA

## Abstract

**Purpose:**

To examine the associations between sentinel lymph node biopsy (SLNB) and complications among older patients who underwent breast-conserving surgery (BCS) for ductal carcinoma in situ (DCIS).

**Methods:**

We identified women from the Surveillance, Epidemiology, and End Results–Medicare dataset aged 67–94 years diagnosed during 1998–2011 with DCIS who underwent BCS as initial treatment. We assessed incidence of complications, including lymphedema, wound infection, seroma, or pain, within 9 months of diagnosis. We used Mahalanobis matching and generalized linear models to estimate the associations between SLNB and complications.

**Results:**

Our sample consisted of 15,515 beneficiaries, 2409 (15.5%) of whom received SLNB. Overall, 16.8% of women who received SLNB had complications, compared with 11.3% of women who did not receive SLNB (*p* < 0.001). Use of SLNB was associated with subsequent mastectomy but not radiotherapy. Multivariate analyses of the matched sample showed that, compared with no SLNB, SLNB use was significantly associated with incidence of any complication [adjusted odds ratio (AOR) 1.39; 99% confidence interval (CI) 1.18–1.63], lymphedema (AOR 4.45; 99% CI 2.27–8.75), wound infection (AOR 1.24; 99% CI 1.00–1.54), seroma (AOR 1.40; 99% CI 1.03–1.91), and pain (AOR 1.31; 99% CI 1.04–1.65). Sensitivity analyses excluding patients who underwent mastectomy yielded qualitatively similar results regarding the associations between SLNB and complications.

**Conclusions:**

Among older women with DCIS who received BCS, SLNB use was associated with higher risks of short-term complications. These findings support consensus guidelines recommending against SLNB for this population and provide empirical information for patients.

**Electronic supplementary material:**

The online version of this article (10.1245/s10434-018-6410-0) contains supplementary material, which is available to authorized users.

With increased use of screening mammography, the incidence of ductal carcinoma in situ (DCIS) has increased dramatically over the past four decades.^1^,^2^ Approximately 55,000 new cases of DCIS occur among US women each year.^3^ Fortunately, breast cancer mortality in patients with pure DCIS remains low, with a reported 10-year cancer-specific survival rate of > 97%.^4^ Treatment of DCIS can include surgery [mastectomy or breast-conserving surgery (BCS)], axillary evaluation, radiotherapy (RT), and endocrine therapy.^5^ Because DCIS itself is rarely fatal, determining the optimal clinical approach to treat DCIS while minimizing complications and side effects is a research priority identified by the Institute of Medicine and the Patient-Centered Outcomes Research Institute.^6^,^7^

Sentinel lymph node biopsy (SLNB) for DCIS management is an area where clinical management can vary. Consensus guidelines, such as those published by the National Comprehensive Cancer Network and the American Society of Clinical Oncology,^8^,^9^ recommend against SLNB in women with DCIS undergoing BCS. However, nearly 17% of patients undergoing BCS for DCIS underwent SLNB.^10^ Furthermore, the findings that SLNB increased from 7.2% in 1998 to 39.4% in 2011 in the USA raised concerns about compliance with these national guidelines.^11^ A 2015 survey in the UK also revealed that surgeons’ view on indications for SLNB differed from national guidelines.^12^ In this procedure, the first axillary node or nodes to drain the breast are identified, removed, and histologically examined. SLNB has replaced axillary lymph node dissection (ALND) for patients with invasive breast cancer and a clinically negative axilla. While less invasive than ALND, SLNB still carries a risk of acute and long-term complications, including lymphedema, wound infection, seroma formation, and pain.^13^ Prior literature evaluating side effects of SLNB was generally limited to the comparison between SLNB and ALND, and to patients with early-stage invasive breast cancer.^14^,^15^ Additionally, some evidence from analyses of invasive breast cancer suggested that older age and advanced stage are associated with increased risk of side effects after surgery.^16^,^17^ However, it is unclear whether SLNB (compared with no SLNB) increases side effects among older patients with DCIS. A recent analysis of nearly 7000 DCIS patients in Sweden found that receipt of SLNB was not associated with decreased risk of breast cancer mortality.^18^ Given the current lack of evidence for SLNB benefit, it is important to determine SLNB-related complications in order to inform treatment decision-making.

This study aimed to examine the association between SLNB and acute complications among female Medicare beneficiaries with DCIS. Older women with DCIS represent a unique group of patients who have very favorable prognosis. While the risks associated with SLNB among women undergoing BCS may be small, we hypothesized that they would be higher than risks without SLNB. We anticipated that SLNB might be associated with more aggressive treatments that also have side effects, such as mastectomy or RT; thus, we controlled for these treatments to determine the independent associations between SLNB and acute complications. Determining higher rates of complications associated with SLNB might potentially discourage SLNB use for patients with DCIS.

## Methods

### Data Source and Study Population

Using the Surveillance, Epidemiology, and End Results (SEER)–Medicare database, we conducted a retrospective cohort study of older female patients diagnosed at age 67–94 years with in situ breast tumors between 1/1/1998 and 12/31/2011.^19^,^20^ We used SEER ICD-O-3 behavior and histology codes to identify patients with DCIS (in situ tumor which is consistent with ductal origin, see Online Appendix Table 1). We only included women who received BCS as first breast surgery in the first 6 months after DCIS diagnosis. Patients were excluded if they were male, their diagnosis occurred only according to death certificate or autopsy, or their income or education by zip code was unknown. Women with SLNB were included in our study if SLNB occurred at any point during their treatment (whether at time of initial lumpectomy, as a separate procedure, or at time of a subsequent mastectomy). The Yale Human Investigation Committee determined that this study did not directly involve human subjects.

### Exposure and Outcome Ascertainment

We identified SLNB according to the Healthcare Common Procedure Coding System codes 38500, 38525, 38790, 38792, 38900, 78195, A9520, G8878 in the first 6 months after DCIS diagnosis based on prior literature^21^–^25^ as well as suggestions from clinicians on our team. Within 9 months from diagnosis we assessed the development of short-term outcomes of lymphedema, wound infection, seroma, or pain (see Online Appendix Table 1). For lymphedema, we included this diagnosis on durable medical equipment claims, as well as the inpatient, outpatient or physician claims assessed for the other short-term outcomes.

### Covariate Selection

Patient characteristics included age at diagnosis, race, marital status, year of DCIS diagnosis, SEER registry, metro status of residence, and comorbidity.^26^ We assessed Elixhauser comorbidity^27^ and created a disability indicator.^28^,^29^ SEER–Medicare also provides census-based estimates of income and education. Tumor characteristics included grade, size, laterality, and estrogen receptor (ER) and progesterone receptor (PR) status, as reported by SEER. We identified flu vaccine, physician visit, and hospitalization as indicators of interaction with the healthcare system in the 3–24 months prior to DCIS diagnosis. Other variables included use of preoperative breast magnetic resonance imaging (MRI), surgeon volume, and receipt of RT. We assessed receipt of mastectomy with and without SLNB after initial BCS through 6 months after DCIS diagnosis.

### Statistical Analysis

We used Mahalanobis matching to adjust for baseline characteristics and account for potential treatment selection bias, where those who receive SLNB might be systematically different from those who do not.^30^,^31^ Matching was based on the calculated Mahalanobis distance, including age, tumor grade, tumor size, hormone receptor status, year of diagnosis (in 2-year groupings), SEER registry, and geographic region. Matches were assigned by choosing the two best non-SLNB patient matches for each SLNB patient; when two or more SLNB patients matched the same control (that is, had Mahalanobis distance minimized by the same control), one was randomly selected as a match, with this process reiterated until nearly all SLNB patients had two matched controls. We assessed balance diagnostics by comparing prevalence of baseline characteristics using absolute standardized differences (expressed as percentage).^32^ Prior research has suggested that two-to-one matching can improve precision,^33^ and standardized difference ≥ 10 indicates meaningful imbalance in the baseline covariate.^34^

We applied generalized linear models to the Mahalanobis matched cohort to estimate the associations of SLNB and complications. In each model, we accounted for the nesting effects within matched groups. We also adjusted for subsequent mastectomy within 180 days after initial BCS, prior MRI, and RT status. The primary outcomes we used in the regression model were occurrence of any complication and occurrence of each individual complication (lymphedema, wound infection, seroma, and pain). To account for multiple comparisons, statistical significance was determined by *p* value lower than 0.01. All statistical analyses in this section were performed using STATA 14 (College Station, TX) and SAS 9.4 (SAS Institute, Cary, NC).

## Results

**Fig. 1:**
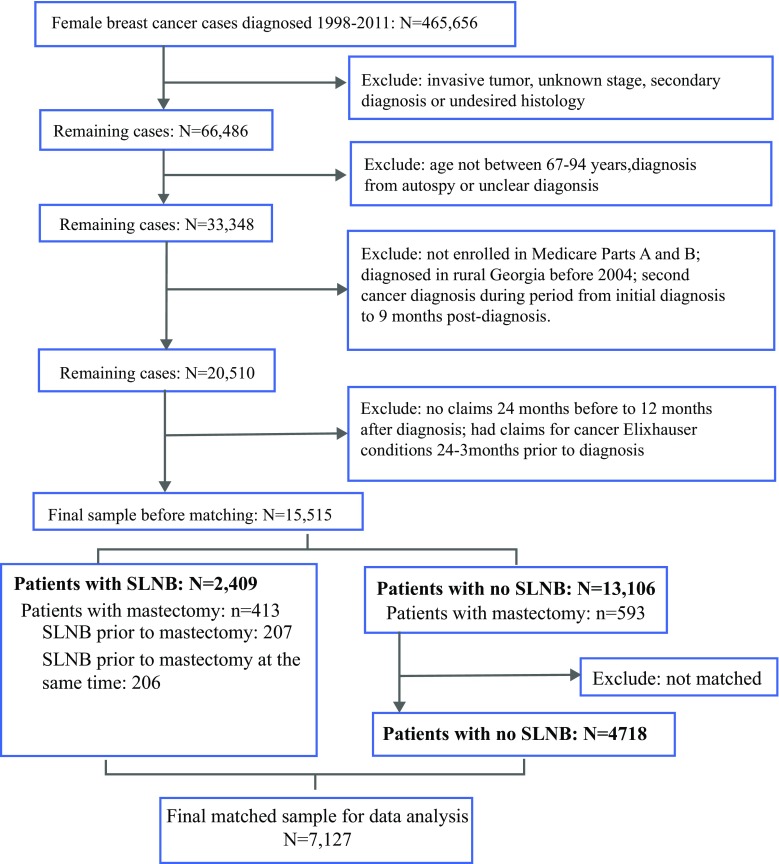
Cohort selection diagram; *SLNB* sentinel lymph node biopsy

The sample consisted of 15,515 women with DCIS (mean age 75.0 years), including 2409 (15.5%) who underwent SLNB and 4718 matched controls. Detailed cohort creation is shown in Fig. 1. Women who underwent SLNB tended to be younger, White, and married, have fewer comorbidities, and be diagnosed in later years (*p* < 0.001 for all except *p* = 0.025 for comorbidity; Table 1). Women who received surgery from surgeons with larger volume were less likely to undergo SLNB (*p* = 0.014). Tumor characteristics, such as high grade, DCIS tumor size > 2 cm, ER-positive DCIS, and comedonecrosis, were associated with the likelihood of undergoing SLNB (*p* < 0.001 for all). All 2409 women undergoing SLNB were successfully matched with 4718 non-SLNB controls (2309 women had two controls, 100 women had one control). After matching, baseline characteristics were well balanced between those who underwent SLNB and those who did not, with standardized differences less than 10. Detailed characteristics are reported in Online Appendix Table 2.

**Table 1:** Selected patient characteristics before and after Mahalanobis matching

	Before match					After match				
	No SLNB		SLNB		SD^a^	No SLNB		SLNB		SD
	*N*	%	*N*	%	%	*N*	%	*N*	%	%
Total sample	13,106	84	2409	16	N/A	4718	66	2409	34	N/A
Age (years)										
67–69	2571	20	573	24	− 10.1	981	21	573	24	− 7.2
70–74	4104	31	783	33	− 2.6	1592	34	783	33	2.6
75–79	3367	26	614	25	0.5	1263	27	614	25	2.9
80–84	2110	16	333	14	6.4	670	14	333	14	1.1
85+	954	7	106	4	12.3	212	4	106	4	0.5
Race										
White	11,369	87	2148	89	− 7.4	4099	87	2148	89	− 7.0
Black	1032	8	170	7	3.1	386	8	170	7	4.2
Other	705	5	91	4	7.7	233	5	91	4	5.7
Hispanic ethnicity										
Yes	545	4	135	6	− 6.7	221	5	135	6	− 4.2
No	12,561	96	2274	94	6.7	4497	95	2274	94	4.2
Marital status										
Married	6211	47	1227	51	− 7.1	2286	48	1227	51	− 5.0
Unmarried	6204	47	1085	45	4.6	2196	47	1085	45	3.0
Other	691	5	97	4	5.9	236	5	97	4	4.7
Grade										
Well differentiated	2048	16	254	11	15.1	498	11	254	11	0.0
Moderately differentiated	4456	34	629	26	17.3	1371	29	629	26	6.6
Poorly differentiated	2925	22	802	33	− 24.7	1449	31	802	33	− 5.5
Undifferentiated	1070	8	321	13	− 16.7	601	13	321	13	− 1.7
Unknown	2607	20	403	17	8.2	799	17	403	17	0.6
Tumor size										
< 2.0 cm	7402	56	1264	52	8.1	2552	54	1264	52	3.2
2.0 to ≤ 5.0 cm	1512	12	434	18	− 18.3	749	16	434	18	− 5.7
> 5.0 cm	179	1	89	4	− 14.9	149	3	89	4	− 2.9
Missing	4013	31	622	26	10.7	1268	27	622	26	2.4
Hormone receptors										
ER– and PR–	923	7	341	14	− 23.3	631	13	341	14	− 2.3
ER+ or PR+	5839	45	1329	55	− 21.4	2608	55	1329	55	0.2
Missing	6344	48	739	31	36.9	1479	31	739	31	1.5
Comedonecrosis										
Yes	1246	10	297	12	− 9.1	524	11	297	12	− 3.8
No	11,860	90	2112	88	9.1	4194	89	2112	88	3.8
Disability										
Yes	410	3	67	3	2.0	129	3	67	3	− 0.3
No	12,696	97	2342	97	− 2.0	4589	97	2342	97	0.3
Elixhauser comorbidity										
None	6321	48	1219	51	− 4.7	2229	47	1219	51	− 6.7
1 to 2	5177	40	935	39	1.4	1914	41	935	39	3.6
3 or more	1608	12	255	11	5.3	575	12	255	11	5.0
Surgeon volume^b^										
1	6243	48	1140	47	0.6	2182	46	1140	47	− 2.2
2	3114	24	578	24	− 0.5	1099	23	578	24	− 1.6
3	1645	13	329	14	− 3.3	596	13	329	14	− 3.0
4+	1865	14	300	12	5.2	727	15	300	12	8.5
Not assigned	239	2	62	3	− 5.1	114	2	62	3	− 1.0
Year of diagnosis										
1998–1999	1039	8	30	1	32.4	63	1	30	1	0.8
2000–2001	1959	15	140	6	30.3	286	6	140	6	1.1
2002–2003	2011	15	255	11	14.2	520	11	255	11	1.4
2004–2005	2163	17	431	18	− 3.7	863	18	431	18	1.0
2006–2007	2021	15	508	21	− 14.7	986	21	508	21	− 0.5
2008–2009	2007	15	539	22	− 18.1	1048	22	539	22	− 0.4
2010–2011	1906	15	506	21	− 17.0	952	20	506	21	− 2.0
Geographic region										
Midwest	1980	15	296	12	8.2	583	12	296	12	0.2
Northeast	3217	25	488	20	10.3	979	21	488	20	1.2
South	2408	18	588	24	− 14.8	1141	24	588	24	− 0.5
West	5501	42	1037	43	− 2.2	2015	43	1037	43	− 0.7

*SLNB* sentinel lymph node biopsy

^a^SD refers to standardized difference, which is a statistic that evaluates the balance of matched cohorts. Standardized difference below 10% indicates balance on the variable

^b^Surgeon volume reflects women who saw a provider who performed BCS on *X* number of women in our sample for the year of this woman’s surgery

### Treatment Received

While BCS was the initial surgery in all patients, 1006 went on to receive completion mastectomy. In the matched cohort, women who underwent SLNB were more likely to receive subsequent mastectomy within 6 months compared with women who did not undergo SLNB (17.1 versus 5.0%, *p* < 0.001; Table 2). Women who underwent SLNB were also more likely to have preoperative MRI examination (20.5 versus 11.5%, *p* < 0.001). There was no statistically significant difference between the two groups in terms of RT receipt (58.5 versus 59.6%, *p* = 0.48). Of the 413 patients who had SLNB and completion mastectomy, 206 women (49.9%) underwent SLNB at time of completion mastectomy and 207 women (50.1%) underwent SLNB prior to mastectomy.

**Table 2:** Treatment received in the sample before and after matching

	Table 2 Before matching					Table 2 After matching				
	No SLNB *N* = 13,106		SLNB *N* = 2409		*χ* ^2^	No SLNB *N* = 4718		SLNB *N* = 2409		*χ* ^2^
	*N*	%	*N*	%	*p* value	*N*	%	*N*	%	*p* value
Preoperative MRI					< 0.001					< 0.001
Yes	1072	8.2	495	20.5		542	11.5	495	20.5	
No	12,034	91.8	1914	79.5		4176	88.5	1914	79.5	
Mastectomy					< 0.001					< 0.001
Yes	593	4.5	413	17.1		237	5.0	413	17.1	
No	12,513	95.5	1996	82.9		4481	95.0	1996	82.9	
Radiotherapy					0.001					0.48
Yes	7237	55.2	1416	58.8		2814	59.6	1416	58.8	
No	5869	44.8	993	41.2		1904	40.4	993	41.2	

*SLNB* sentinel lymph node biopsy, *MRI* magnetic resonance imaging

### Acute Complications

In the matched sample, SLNB was associated with increased risk of acute complications (Table 3). Occurrence of any complication was 16.8% in the SLNB group and 11.3% in the non-SLNB group (*p* < 0.001). Multivariate models revealed that SLNB use was independently associated with increased risk of complications (Table 4). Women who underwent SLNB had significantly higher risk of any complication [adjusted odds ratio (AOR) 1.39; 99% confidence interval (CI) 1.18–1.63]. Specifically, SLNB use was associated with each complication, including lymphedema (AOR 4.45; 99% CI 2.27–8.75), wound infection (AOR 1.24; 99% CI 1.00–1.54), seroma (AOR 1.40; 99% CI 1.03–1.91), and pain (AOR 1.31; 99% CI 1.04–1.65). Mastectomy was associated with increased risk of any complication (AOR 1.36; 99% CI 1.05–1.77), wound infection (AOR 1.55; 99% CI 1.12–2.45), and seroma (AOR 2.51; 99% CI 1.60–3.92). Prior MRI use and RT were not significantly associated with acute complications. Sensitivity analyses using the sample before matching and including or excluding patients who underwent mastectomy reached qualitatively similar results regarding the associations between SLNB and complications.

**Table 3:** Unadjusted side effects in the sample before and after matching

	Table 2 Before matching					Table 2 After matching				
	No SLNB *N* = 13,106		SLNB *N* = 2409		*χ* ^2^	No SLNB *N* = 4718		SLNB *N* = 2409		*χ* ^2^
	*N*	%	*N*	%	*p* value	*N*	%	*N*	%	*p* value
Any of below	1476	11.3	404	16.8	< 0.001	534	11.3	404	16.8	< 0.001
Lymphedema	57	0.4	60	2.5	< 0.001	23	0.5	60	2.5	< 0.001
Wound infection	1290	9.8	296	12.3	< 0.001	453	9.6	296	12.3	< 0.001
Seroma	421	3.2	153	6.4	< 0.001	179	3.8	153	6.4	< 0.001
Pain	1060	8.1	237	9.8	0.004	365	7.7	237	9.8	0.003

*SLNB* sentinel lymph node biopsy

**Table 4:** Generalized models for the association of sentinel lymph node biopsy (SLNB) with specified outcome

Model description	Any complication	Lymphedema	Wound infection	Seroma	Pain
SLNB					
No	REF	REF	REF	REF	REF
Yes	1.39 (1.18–1.63)	4.45 (2.27–8.75)	1.24 (1.00–1.54)	1.40 (1.03–1.91)	1.31 (1.04–1.65)
Mastectomy after BCS					
No	REF	REF	REF	REF	REF
Yes	1.36 (1.05–1.77)	2.17 (0.98–4.82)	1.55 (1.12–2.15)	2.51 (1.60–3.92)	0.88 (0.58–1.33)
Radiotherapy					
No	REF	REF	REF	REF	REF
Yes	0.85 (0.85–1.31)	0.86 (0.44–1.68)	0.81 (0.65–1.02)	1.07 (0.76–1.51)	0.85 (0.67–1.07)
Prior MRI					
No	REF	REF	REF	REF	REF
Yes	1.05 (0.72–1.00)	1.30 (0.64–2.61)	0.94 (0.70–1.25)	1.82 (1.27–2.60)	1.06 (0.78–1.44)

Accounting for matching and adjusting for treatment received. Adjusted odds ratio (99% confidence interval)

*MRI* magnetic resonance imaging, *BCS* breast-conserving surgery

## Discussion

Among women with DCIS who received BCS as initial surgery, use of SLNB was significantly associated with increased risk of complications, including lymphedema, wound infection, seroma, and pain. Given the lack of evidence that SLNB improves long-term outcomes for patients with DCIS,^35^ our finding of increased risk of SLNB-related complications should further discourage its routine use in these patients.

Our study advances current knowledge about SLNB use in DCIS patients in several important ways. First, we compared use of SLNB versus no SLNB in real-world practice. Existing studies, generally performed as part of a clinical trial or at individual institutions, have demonstrated that SLNB for invasive breast cancer was less likely to lead to side effects compared with ALND, a more invasive procedure.^36^ For instance, a 2015 review showed that the incidence of lymphedema after ALND was 22.3% and the incidence after SLNB was 6.3%.^36^ While the incidence of SLNB-related complications is generally acceptable, population-level data on side effects of SLNB and comparisons between SLNB and no SLNB, specifically among patients with DCIS, are lacking. This study, to the best of the authors’ knowledge, is the first to fill this evidence gap. Our findings reveal that approximately one out of six patients who underwent BCS plus SLNB would experience complications compared with one out of nine patients who underwent BCS without SLNB. The latter cohort of patients who experienced complications without axillary surgery merit further study. Our findings support the consensus guidelines that SLNB should not be routinely used for DCIS patients who undergo BCS.

Second, the odds ratio of lymphedema attributed to SLNB use is relatively large, estimated at 4.41. This information is important because both the risk of developing lymphedema and the associated symptoms often persist over the course of a woman’s lifetime and therefore have a great impact on quality of life.^37^,^38^ We found that the incidence of lymphedema among women who underwent SLNB was only 2.5%, relatively low compared with prior literature.^36^ We limited our study to patients with DCIS, which has low likelihood of nodal involvement. The sentinel node procedure includes removal of not only “hot” or “blue” nodes, but also any clinically suspicious nodes. It may be that, during SLNB for DCIS, the clinical suspicion of the surgeon is lower, leading to resection of fewer lymph nodes in this setting, and a resultant lower rate of lymphedema than seen in other series. Additionally, we used claims data to identify lymphedema occurrence; such an approach might only capture severe lymphedema and thus underestimate the incidence. Nevertheless, our results indicate that the incidence of having a claim for lymphedema (0.5%) was very low for patients who did not undergo SLNB, and use of SLNB is a strong predictor for this complication.

Analyzing data from over 600 acute-care hospitals throughout the USA, a prior study found that 16.7% of patients who received BCS for DCIS underwent SLNB and surgeons who had low patient volume were more likely to perform SLNB.^10^ Building upon this study, our findings suggest that, in addition to low surgeon volume, tumor characteristics, including grade, size, ER status, and comedonecrosis, are also associated with SLNB use. Furthermore, we found that patients who underwent SLNB were more likely to receive preoperative MRI and subsequent mastectomy, reflecting the fact that physicians who are more aggressive with respect to axillary surgery may also be more aggressive with respect to imaging and extent of breast surgery. It is also possible that less experienced surgeons tended to use more aggressive treatments such as SLNB and mastectomy. While SLNB use at time of mastectomy for patients with DCIS might be appropriate, the proportion was quite low in our cohort. In fact, more than 90% of SLNB use was performed outside of current recommendations, either before mastectomy or without undergoing mastectomy. These findings are important, particularly in the context of current controversies regarding overdiagnosis and overtreatment of DCIS patients.^39^,^40^ Future programs targeting low-volume physicians with practice improvement interventions may improve DCIS care quality and reduce inappropriate care for patients with DCIS.

Our study, however, has some limitations. First, we only examined short-term SLNB-related complications. Future research examining long-term side effects, recurrence, and survival attributed to SLNB is needed. Second, our study was limited to older population; thus, our results should not be generalized to younger population. While our population comprised beneficiaries enrolled in Medicare fee-for-service programs, we would be surprised if the harm attributed to SLNB differed among Medicare Part C beneficiaries. Third, we used medical claims to capture side effects, including pain, which are subject to estimation errors. Finally, our study is not a randomized trial. While we applied a Mahalanobis matching method to reduce selection bias, we were unable to control for unobserved factors. For instance, obesity is associated with lymphedema,^16^ yet we do not have data of individual body mass index.

In conclusion, SLNB use led to higher rate of short-term side effects among women undergoing BCS for DCIS. Given a lack of evidence that SLNB use decreases recurrence or improves breast cancer survival for patients with DCIS, our results indicate that using SLNB to detect nodal involvement may be causing more harm than good for older women with DCIS. These data highlight the need to critically consider the impact of SLNB on patient health outcomes.

## Electronic supplementary material

Below is the link to the electronic supplementary material.

Supplementary material 1 (DOCX 29 kb)
