# Supplementary material for: Associations Between Sentinel Lymph Node Biopsy and Complications for Patients with Ductal Carcinoma In Situ
Source: Ann Surg Oncol. 2018 Mar 7;25(6):1521–9. doi: 10.1245/s10434-018-6410-0 (PMC5928184; doi:10.1245/s10434-018-6410-0)
Supplement: Supplementary file 1 — Supplementary material 1 (DOCX 29 kb) [file 10434_2018_6410_MOESM1_ESM.docx]

**Supplementary materials**

**Appendix Table 1. Administrative codes used for analysis**

| ICD-O-3 Histology | Include: 8000, 8001, 8010, 8020, 8021, 8022, 8050, 8140, 8201, 8211, 8230, 8255, 8260, 8480, 8481, 8490, 8500, 8501, 8502, 8503, 8504, 8507, 8510, 8512, 8513, 8514, 8521, 8522, 8523, 8525, 8530, 8540, 8541, 8543, 8570, 8571, 8572, 8573, 8574, 8575 Exclude: 8520, 8524 |
| --- | --- |
| Breast Conserving Surgery | HCPCS: 19110, 19120, 19125, 19126, 19160, 19162, 19301, 19302 ICD-9 Procedure: 85.20-85.23, 85.25 |
| Mastectomy | HCPCS: 19180, 19182, 19200, 19220, 19240, 19303, 19304, 19305, 19306, 19307 ICD-9 Procedure: 85.41-48 |
| Sentinel Lymph Node Biopsy | HCPCS: 38500, 38525, 38790, 38792, 38900, 78195, A9520, G8878 |
| MRI | HCPCS: 76093, 76094, 77058, 77059, C8903-C8908 |
| Lymphedema | ICD-9 diagnosis: 457.0 or 457.1 |
| Wound infection | Wound complications  HCPCS: 10121, 11000, 11001, 11040-11044, 12020, 12021, 13160, 97597, 97598, 97601, 97602, 97605, 97606, 99183  ICD-9 Procedure: 86.22, 86.28, 93.59, 93.95, 96.59, 97.16  ICD-9 diagnosis: 709.4, 998.6, 998.83, 998.1x, 998.3x |
|  | Infection  HCPCS: 10060, 10061, 10160, 10180  ICD-9 diagnosis: 041.9, 682.9, 999.31, 998.5x |
|  | Severe infection  ICD-9 diagnosis: 785.59, 790.7, 999.3, 038.x, 785.5x |
| Seroma | ICD-9 diagnosis: 998.13 |
| Pain | Breast pain  ICD-9 diagnosis: 611.71 |
|  | Limitation of movement of upper extremities  ICD-9 diagnosis: 719.51-52 or 719.91-92 |
|  | Paresthesia  ICD-9 diagnosis: 782.0 |

**Appendix Table 2:** Patient characteristics before and after Mahalanobis matching

|  | Before Match | | | | | After Match | | | | |
| --- | --- | --- | --- | --- | --- | --- | --- | --- | --- | --- |
|  | No SLNB | | SLNB | | St Dif* | No SLNB | | SLNB | | St Dif |
|  | N | % | N | % | % | N | % | N | % | % |
| Total Sample | 13,106 | 84% | 2,409 | 16% | N/A | 4,718 | 66% | 2,409 | 34% | N/A |
| Age |  |  |  |  |  |  |  |  |  |  |
| 67-69 | 2,571 | 20% | 573 | 24% | -10.1% | 981 | 21% | 573 | 24% | -7.2% |
| 70-74 | 4,104 | 31% | 783 | 33% | -2.6% | 1,592 | 34% | 783 | 33% | 2.6% |
| 75-79 | 3,367 | 26% | 614 | 25% | 0.5% | 1,263 | 27% | 614 | 25% | 2.9% |
| 80-84 | 2,110 | 16% | 333 | 14% | 6.4% | 670 | 14% | 333 | 14% | 1.1% |
| 85+ | 954 | 7% | 106 | 4% | 12.3% | 212 | 4% | 106 | 4% | 0.5% |
| Race† |  |  |  |  |  |  |  |  |  |  |
| White | 11,369 | 87% | 2,148 | 89% | -7.4% | 4,099 | 87% | 2,148 | 89% | -7.0% |
| Black | 1,032 | 8% | 170 | 7% | 3.1% | 386 | 8% | 170 | 7% | 4.2% |
| Other | 705 | 5% | 91 | 4% | 7.7% | 233 | 5% | 91 | 4% | 5.7% |
| Hispanic Ethnicity |  |  |  |  |  |  |  |  |  |  |
| Yes | 545 | 4% | 135 | 6% | -6.7% | 221 | 5% | 135 | 6% | -4.2% |
| No | 12,561 | 96% | 2,274 | 94% | 6.7% | 4,497 | 95% | 2,274 | 94% | 4.2% |
| Marital Status |  |  |  |  |  |  |  |  |  |  |
| Married | 6,211 | 47% | 1,227 | 51% | -7.1% | 2,286 | 48% | 1,227 | 51% | -5.0% |
| Unmarried | 6,204 | 47% | 1,085 | 45% | 4.6% | 2,196 | 47% | 1,085 | 45% | 3.0% |
| Other | 691 | 5% | 97 | 4% | 5.9% | 236 | 5% | 97 | 4% | 4.7% |
| Grade |  |  |  |  |  |  |  |  |  |  |
| Well differentiated | 2,048 | 16% | 254 | 11% | 15.1% | 498 | 11% | 254 | 11% | 0.0% |
| Moderately differentiated | 4,456 | 34% | 629 | 26% | 17.3% | 1,371 | 29% | 629 | 26% | 6.6% |
| Poorly differentiated | 2,925 | 22% | 802 | 33% | -24.7% | 1,449 | 31% | 802 | 33% | -5.5% |
| Undifferentiated | 1,070 | 8% | 321 | 13% | -16.7% | 601 | 13% | 321 | 13% | -1.7% |
| Unknown | 2,607 | 20% | 403 | 17% | 8.2% | 799 | 17% | 403 | 17% | 0.6% |
| Tumor size |  |  |  |  |  |  |  |  |  |  |
| <2.0 cm | 7,402 | 56% | 1,264 | 52% | 8.1% | 2,552 | 54% | 1,264 | 52% | 3.2% |
| 2.0-<=5.0 cm | 1,512 | 12% | 434 | 18% | -18.3% | 749 | 16% | 434 | 18% | -5.7% |
| >5.0 cm | 179 | 1% | 89 | 4% | -14.9% | 149 | 3% | 89 | 4% | -2.9% |
| Missing | 4,013 | 31% | 622 | 26% | 10.7% | 1,268 | 27% | 622 | 26% | 2.4% |
| Laterality |  |  |  |  |  |  |  |  |  |  |
| Right | 6,466 | 49% | 1,154 | 48% | 2.9% | 2,330 | 49% | 1,154 | 48% | 3.0% |
| Left | 6,640 | 51% | 1,255 | 52% | -2.9% | 2,388 | 51% | 1,255 | 52% | -3.0% |
| Hormone receptors |  |  |  |  |  |  |  |  |  |  |
| ER– and PR– | 923 | 7% | 341 | 14% | -23.3% | 631 | 13% | 341 | 14% | -2.3% |
| ER+ or PR+ | 5,839 | 45% | 1,329 | 55% | -21.4% | 2,608 | 55% | 1,329 | 55% | 0.2% |
| Missing | 6,344 | 48% | 739 | 31% | 36.9% | 1,479 | 31% | 739 | 31% | 1.5% |
| Comedonecrosis |  |  |  |  |  |  |  |  |  |  |
| Yes | 1,246 | 10% | 297 | 12% | -9.1% | 524 | 11% | 297 | 12% | -3.8% |
| No | 11,860 | 90% | 2,112 | 88% | 9.1% | 4,194 | 89% | 2,112 | 88% | 3.8% |
| Disability |  |  |  |  |  |  |  |  |  |  |
| Yes | 410 | 3% | 67 | 3% | 2.0% | 129 | 3% | 67 | 3% | -0.3% |
| No | 12,696 | 97% | 2,342 | 97% | -2.0% | 4,589 | 97% | 2,342 | 97% | 0.3% |

**Appendix Table 2:** Patient characteristics before and after Mahalanobis matching (continued)

|  | Before Match | | | | | After Match | | | | |
| --- | --- | --- | --- | --- | --- | --- | --- | --- | --- | --- |
|  | No SLNB | | SLNB | | St. Dif | No SLNB | | SLNB | | St. Dif |
|  | N | % | N | % | % | N | % | N | % | % |
| Elixhauser Comorbidity |  |  |  |  |  |  |  |  |  |  |
| None | 6,321 | 48% | 1,219 | 51% | -4.7% | 2,229 | 47% | 1,219 | 51% | -6.7% |
| 1 to 2 | 5,177 | 40% | 935 | 39% | 1.4% | 1,914 | 41% | 935 | 39% | 3.6% |
| 3 or more | 1,608 | 12% | 255 | 11% | 5.3% | 575 | 12% | 255 | 11% | 5.0% |
| Medicaid coverage (12 months pre-dx) |  |  |  |  |  |  |  |  |  |  |
| Yes | 1,266 | 10% | 197 | 8% | 5.2% | 482 | 10% | 197 | 8% | 7.1% |
| No | 11,840 | 90% | 2,212 | 92% | -5.2% | 4,236 | 90% | 2,212 | 92% | -7.1% |
| Median income of census tract or zip code |  |  |  |  |  |  |  |  |  |  |
| Less than $33,000 | 2,242 | 17% | 392 | 16% | 2.2% | 892 | 19% | 392 | 16% | 6.9% |
| $33,000-40,000 | 1,715 | 13% | 350 | 15% | -4.2% | 655 | 14% | 350 | 15% | -1.9% |
| $40,000-50,000 | 2,586 | 20% | 499 | 21% | -2.4% | 942 | 20% | 499 | 21% | -1.9% |
| $50,000-63,000 | 2,784 | 21% | 507 | 21% | 0.5% | 920 | 19% | 507 | 21% | -3.8% |
| More than $63,000 | 3,779 | 29% | 661 | 27% | 3.1% | 1,309 | 28% | 661 | 27% | 0.7% |
| Percent HS education or less of census tract or zip code |  |  |  |  |  |  |  |  |  |  |
| <30 % | 3,857 | 29% | 700 | 29% | 0.8% | 1,347 | 29% | 700 | 29% | -1.1% |
| 30 to < 40% | 2,344 | 18% | 436 | 18% | -0.6% | 836 | 18% | 436 | 18% | -1.0% |
| 40 to <50 % | 2,330 | 18% | 419 | 17% | 1.0% | 801 | 17% | 419 | 17% | -1.1% |
| 50 to < 60% | 2,154 | 16% | 368 | 15% | 3.2% | 788 | 17% | 368 | 15% | 3.9% |
| <= 60% | 2,421 | 18% | 486 | 20% | -4.3% | 946 | 20% | 486 | 20% | -0.3% |
| County Metropolitan Status |  |  |  |  |  |  |  |  |  |  |
| Metro | 11,446 | 87% | 2,089 | 87% | 1.8% | 3,998 | 85% | 2,089 | 87% | -5.7% |
| Nonmetro | 1,660 | 13% | 320 | 13% | -1.8% | 720 | 15% | 320 | 13% | 5.7% |
| Flu Vaccine (24-3 months prior to diagnosis) |  |  |  |  |  |  |  |  |  |  |
| Yes | 8,700 | 66% | 1,574 | 65% | 2.2% | 3,132 | 66% | 1,574 | 65% | 2.2% |
| No | 4,406 | 34% | 835 | 35% | -2.2% | 1,586 | 34% | 835 | 35% | -2.2% |
| Physician Visit (24-3 months prior to diagnosis) |  |  |  |  |  |  |  |  |  |  |
| Yes | 12,794 | 98% | 2,354 | 98% | -0.6% | 4,614 | 98% | 2,354 | 98% | 0.5% |
| No | 312 | 2% | 55 | 2% | 0.6% | 104 | 2% | 55 | 2% | -0.5% |
| Hospitalization (24-3 months prior to diagnosis) |  |  |  |  |  |  |  |  |  |  |
| Yes | 2,597 | 20% | 450 | 19% | 2.9% | 917 | 19% | 450 | 19% | 1.9% |
| No | 10,509 | 80% | 1,959 | 81% | -2.9% | 3,801 | 81% | 1,959 | 81% | -1.9% |
| Surgeon Volume‡ |  |  |  |  |  |  |  |  |  |  |
| 1 | 6,243 | 48% | 1,140 | 47% | 0.6% | 2,182 | 46% | 1,140 | 47% | -2.2% |
| 2 | 3,114 | 24% | 578 | 24% | -0.5% | 1,099 | 23% | 578 | 24% | -1.6% |
| 3 | 1,645 | 13% | 329 | 14% | -3.3% | 596 | 13% | 329 | 14% | -3.0% |
| 4+ | 1,865 | 14% | 300 | 12% | 5.2% | 727 | 15% | 300 | 12% | 8.5% |
| Surgeon not assigned | 239 | 2% | 62 | 3% | -5.1% | 114 | 2% | 62 | 3% | -1.0% |

**Appendix Table 2:** Patient characteristics before and after Mahalanobis matching (continued)

|  | Before Match | | | | | After Match | | | | |
| --- | --- | --- | --- | --- | --- | --- | --- | --- | --- | --- |
|  | No SLNB | | SLNB | | St. Dif | No SLNB | | SLNB | | St. Dif |
|  | N | % | N | % | % | N | % | N | % | % |
| Year of Diagnosis |  |  |  |  |  |  |  |  |  |  |
| 1998-1999 | 1,039 | 8% | 30 | 1% | 32.4% | 63 | 1% | 30 | 1% | 0.8% |
| 2000-2001 | 1,959 | 15% | 140 | 6% | 30.3% | 286 | 6% | 140 | 6% | 1.1% |
| 2002-2003 | 2,011 | 15% | 255 | 11% | 14.2% | 520 | 11% | 255 | 11% | 1.4% |
| 2004-2005 | 2,163 | 17% | 431 | 18% | -3.7% | 863 | 18% | 431 | 18% | 1.0% |
| 2006-2007 | 2,021 | 15% | 508 | 21% | -14.7% | 986 | 21% | 508 | 21% | -0.5% |
| 2008-2009 | 2,007 | 15% | 539 | 22% | -18.1% | 1,048 | 22% | 539 | 22% | -0.4% |
| 2010-2011 | 1,906 | 15% | 506 | 21% | -17.0% | 952 | 20% | 506 | 21% | -2.0% |
| SEER Registry |  |  |  |  |  |  |  |  |  |  |
| Connecticut | 1,217 | 9% | 143 | 6% | 12.7% | 292 | 6% | 143 | 6% | 1.1% |
| Detroit | 1,216 | 9% | 128 | 5% | 15.3% | 259 | 5% | 128 | 5% | 0.8% |
| Hawaii | 241 | 2% | 16 | 1% | 10.6% | 32 | 1% | 16 | 1% | 0.2% |
| Iowa | 764 | 6% | 168 | 7% | -4.7% | 324 | 7% | 168 | 7% | -0.4% |
| New Mexico | 232 | 2% | 49 | 2% | -1.9% | 98 | 2% | 49 | 2% | 0.3% |
| Seattle | 836 | 6% | 144 | 6% | 1.7% | 283 | 6% | 144 | 6% | 0.1% |
| Utah | 304 | 2% | 46 | 2% | 2.9% | 85 | 2% | 46 | 2% | -0.8% |
| Kentucky | 649 | 5% | 184 | 8% | -11.1% | 351 | 7% | 184 | 8% | -0.8% |
| Louisiana | 596 | 5% | 176 | 7% | -11.7% | 332 | 7% | 176 | 7% | -1.0% |
| New Jersey | 2,000 | 15% | 345 | 14% | 2.6% | 687 | 15% | 345 | 14% | 0.7% |
| All Georgia Registries | 1,163 | 9% | 228 | 9% | -2.0% | 458 | 10% | 228 | 9% | 0.8% |
| All California Registries | 3,888 | 30% | 782 | 32% | -6.0% | 1,517 | 32% | 782 | 32% | -0.7% |
| Geographic Region |  |  |  |  |  |  |  |  |  |  |
| Midwest | 1,980 | 15% | 296 | 12% | 8.2% | 583 | 12% | 296 | 12% | 0.2% |
| Northeast | 3,217 | 25% | 488 | 20% | 10.3% | 979 | 21% | 488 | 20% | 1.2% |
| South | 2,408 | 18% | 588 | 24% | -14.8% | 1,141 | 24% | 588 | 24% | -0.5% |
| West | 5,501 | 42% | 1,037 | 43% | -2.2% | 2,015 | 43% | 1,037 | 43% | -0.7% |

*St. Dif refers to standardized difference, which is a statistic that evaluates the balance of matched cohorts. A standardized difference below 10% indicates balance on the variable.

†People with Hispanic ethnicity may be included in any category.

‡ Surgeon volume- reflects women who saw a provider who performed BCS on X number of women in our sample for the year of this woman’s surgery.

SLNB: Sentinel lymph node biopsy.
